# Opioid withdrawal syndrome induced by naldemedine administration in a cancer patient without brain metastasis

**DOI:** 10.1017/S147895152500001X

**Published:** 2025-03-13

**Authors:** Mayumi Ishida, Kojun Okamoto, Isamu Koyama, Nozomu Uchida, Izumi Sato, Akira Yoshioka, Ryota Sato, Hideki Onishi

**Affiliations:** 1 Department of Psycho-Oncology, Saitama Medical University International Medical Center, Saitama, Japan; 2 Department of Hepatobiliary & Pancreatic Surgery, Saitama Medical University International Medical Center, Saitama, Japan; 3 Department of Palliative Medicine, Saitama Medical University International Medical Center, Saitama, Japan; 4 Department of Clinical Epidemiology, Graduate School of Biomedical Sciences, Nagasaki University, Nagasaki, Japan; 5 Department of Medical Oncology and Palliative Care, Mitsubishi Kyoto Hospital, Kyoto, Japan; 6 Department of Pharmacy, Maruki Memorial Medical and Social Welfare Center, Saitama, Japan; 7 Departments of Psycho-oncology, Saitama Medical University International Medical Center, Saitama, Japan

**Keywords:** Naldemedine, opioid withdrawal syndrome, brain metastasis, psychiatric symptoms, cancer

## Abstract

**Objectives:**

Naldemedine is a peripherally acting μ-opioid receptor antagonist used to treat opioid-induced constipation. As this drug does not cross the blood–brain barrier, it is believed that patients without brain metastases do not experience opioid withdrawal symptoms.

**Methods:**

Here, we experienced a case in which a cancer patient without brain metastasis presented with anxiety and restlessness that was severe enough to interfere with daily life. The patient was diagnosed with naldemedine-induced opioid withdrawal syndrome.

**Results:**

The patient was a 66-year-old male with liver cancer metastasizing to the chest wall, but without brain metastasis. Oxycodone was started at 10 mg/day 2 months prior to his visit to our department to treat pain related to the chest wall metastasis, and was increased to 100 mg/day 1 month later and maintained at that dose. Naldemedine was administered as a countermeasure against opioid-induced constipation. The patient developed anxiety and restlessness 10 days prior to his initial visit to our department.

After detailed examination, naldemedine-related opiod withdrawal syndrome was suspected on the basis of anxiety, agitation, and episodes of sudden onset sweating, and these symptoms disappeared within 2 days after the discontinuation of naldemedine, with no recurrence observed thereafter. In addition, head MRI revealed no brain metastasis.

**Significance of the results:**

Even in patients without brain metastasis, naldemedine can induce opioid withdrawal symptoms, so caution is required with patients receiving this drug. In addition, when psychiatric symptoms are pronounced, as in this case, withdrawal symptoms may be underdiagnosed.

## Introduction

Constipation is a frequent side effect of opioid use, and leads to a decline in quality of life (Mesía et al. 2019). Naldemedine, a peripherally acting μ-opioid receptor antagonist, has been used for the treatment of such constipation. As naldemedine has a large molecular weight, it does not pass through the blood–brain barrier and does not act on central μ-opioid receptors. Thus, it was thought that it would not cause opioid withdrawal syndrome (OWS) (Katakami et al. 2017; Webster et al. 2018); however, cases of OWS occurring immediately after the administration of naldemedine have been reported in patients without brain metastasis (Ishida et al. 2022a; Ishii et al. 2020).

In cases where OWS occurred immediately after naldemedine administration, significant physical and psychological symptoms were also observed immediately after administration, clearly indicating a causal relationship with the drug. On the other hand, there have been reports of cases in which the onset of symptoms occurred more than a month after administration (Ishida et al. 2022b; Sato et al. 2023), and it is difficult to identify such symptoms as OWS due to their being mainly psychiatric in nature. Therefore, it is possible for OWS to be overlooked in patients without brain metastasis under such conditions, and it is necessary to accumulate more cases.

Here, we report a case in which the patient developed anxiety and restlessness that worsened at night and was diagnosed with OWS through detailed clinical observation. The symptoms quickly disappeared after discontinuation of naldemedine administration.

## Case report

The patient was a 66-year-old male with a history of hepatitis C.

**Figure 1.**
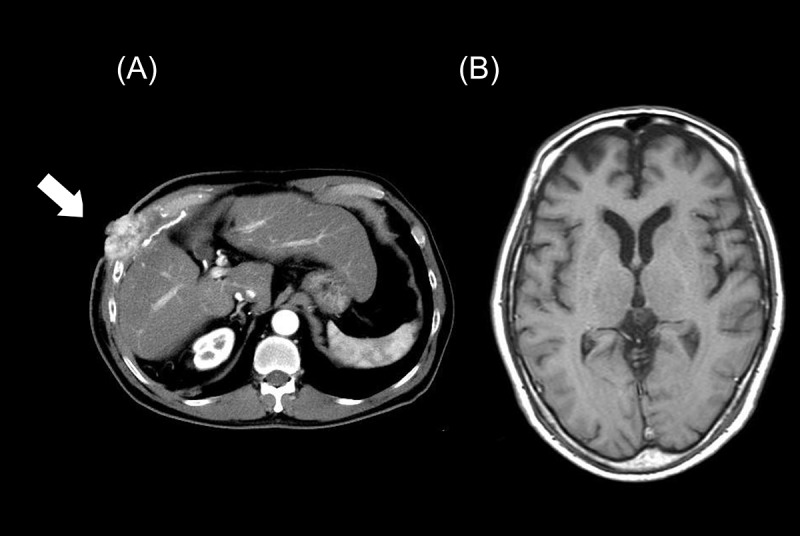
Patient imaging findings. (A) Abdominal CT findings. The white arrow indicates chest wall metastasis. (B) Head MRI findings. No brain metastases were observed.

He was diagnosed with liver cancer 5 years previously and underwent partial liver resection 4 years ago. Three years ago, metastasis to the right chest wall appeared (Figure 1(A)), and surgical resection followed by radiotherapy was performed. Lenvatinib (Kudo et al. 2018) was started 4 months prior to his visit to our department. However, as pain developed in the same area and began to interfere with his activities of daily life, oxycodone 10 mg/day was prescribed to control pain 2 months prior to his visit. Naldemedine was also administered as a countermeasure against opioid-induced constipation as no brain metastasis was observed. Thereafter, the dose of oxycodone was increased to 100 mg/day and the pain disappeared.

The patient suddenly started to feel anxious and restless, which lasted for 10 days. He reported the symptoms to his oncologist, who referred him to the Department of Psycho-oncology.

At his initial consultation, he reported palpitations, exhaustion, and feelings of fatigue in his lower limbs, and that it was painful to sit still. In addition, from a psychological perspective, he complained of symptoms such as increased anxiety at night and, as he could not calm down, he sometimes became violent toward his wife.

According to the patient’s wife, he was originally a very calm person, but he began to become irritable from 10 days prior to his visit to our department, and was unable to sit still and walked around the house claiming, “I’ve done something terrible,” “I feel like I’m going to lose control,” and “I feel overwhelmed, like I can’t control myself.”

There was no impaired consciousness, and no depressive mood or loss or motivation that met the criteria for depression.

Physical findings included a body temperature of 36.7℃, blood pressure of 111/78 mmHg, and a pulse rate of 98/min. Information was also obtained regarding episodes of sudden onset sweating.

The patient’s complaints centered around psychiatric symptoms such as anxiety and irritability that had appeared suddenly 10 days previously and increased at night. There was no evidence of delirium or depression at the time of examination or based on comments from his family. Furthermore, no drugs that could induce akathisia were being administered. As the patient was receiving opioids and naldemedine, we suspected OWS.

We next asked his wife about his objective symptoms at home and she mentioned that he suddenly started sweating profusely, especially from the back of his head.

We therefore checked the degree of OWS using the Clinical Opiate Withdrawal Score (COWS). His score of 10 points suggested the possibility of mild-OWS (Table 1), and treatment with naldemedine, a drug known to interact with opioid receptors, was discontinued.

**Table 1.** The COWS before and after discontinuation of naldemedine

Item	Before discontinuation	Two days after discontinuation
Resting pulse rate	1	1
GI Upset: over last 1/2 hour	0	0
Sweating: Over past 1/2 hour not accounted for by room temperature or patient activity.	3	0
Tremor *Observation of outstretched hands*	0	0
Restlessness *Observation during assessment*	1	0
Yawning *Observation during assessment*	0	0
Pupil size	0	0
Anxiety or irritability	3	0
Bone or joint aches	2	0
Gooseflesh skin	0	0
Runny nose or tearing	0	0
Total score	10	1

Score: 5–12 = mild; 13–24 = moderate; 25–36 = moderately severe; more than 36 = severe withdrawal.

The day after discontinuing naldemedine administration, the patient experienced anxiety in the morning only, and 2 days after discontinuing administration, his COWS score was 1 point (Table 1). No similar symptoms have been observed for over a year since discontinuation. As the patient developed OWS, the possibility of brain metastasis was considered and a head MRI was also performed, but no brain metastasis was detected (Figure 1(B)).

This case was diagnosed as naldemedine-induced OWS based on the clinical symptoms and the course after naldemedine administration.

## Discussion

We experienced a case in which OWS developed 2 months after the start of naldemedine administration in a patient without brain metastasis. The main symptoms were psychiatric in nature, and OWS was diagnosed after detailed observation. On discontinuation of naldemedine, the patient showed a complete recovery from OWS and the accompanying psychiatric symptoms.

It is worth noting that the patient did not have brain metastasis and his chief complaint consisted of psychiatric symptoms. As pointed out by the FDA, naldemedine is not recommended for patients with possible disruptions to the blood-brain barrier (Food and Drug Administration 2017), so opioid withdrawal symptoms due to the relationship between naldemedine and the blood-–brain barrier are rarely suspected at first, and such symptoms are more likely to be considered psychiatric symptoms.

The symptom that helped in the diagnosis was the episodes of sudden onset sweating. Opioid withdrawal has been diagnosed as a disease characterized by the sudden onset of sweating, and cases of withdrawal from naldemedine have been reported in previous studies (Ishida et al. 2022b; Sato et al. 2023), leading to a clinical diagnosis.

In this case, the treatment for OWS consisted of the discontinuation of naldemedine rather that the administration of an opioid antagonist. The reasons for this approach were that the patient’s opioid dosage had not been reduced during the course of treatment, and symptoms suggestive of OWS appeared after the administration of naldemedine. It was hypothesized that, for some reason, naldemedine had passed through the blood–brain barrier and acted on the central μ-opioid receptors, functioning as an opioid antagonist and thereby triggering OWS.

Discontinuation of naldemedine was, therefore, prioritized over the administration of an opioid agonist. Furthermore, as there was no subsequent worsening of pain, no additional opioid agonist was administered.

The symptoms of OWS vary (Kosten and Baxter 2019), are nondisease specific, and resemble the various symptoms observed in patients with advanced cancer (Walsh et al. 2000). Therefore, in cases such as that presented herein where the opioid dose is not reduced or discontinued and the patient’s symptoms are primarily psychiatric, opioid withdrawal may be overlooked. Additionally, sweating was not included as one of the patient’s chief complaints, but was information obtained from his wife and subsequently confirmed through medical evaluation. If one is not aware that opioid withdrawal can occur with naldemedine administration, even in patients without brain metastasis, it is very easy to overlook.

Opioid withdrawal has not been reported in relation to naldemedine in research or clinical practice (Katakami et al. 2017; Naya et al. 2023), moreover it has been reported that, regardless of potential brain metastasis, naldemedine was found to not reduce the analgesic effect of opioids or induce OWS (Osaka et al. 2019). However, there have been cases in which patients without brain metastasis experience opioid withdrawal (Ishida et al. 2022a; Ishii et al. 2020). In light of these multiple reports, it is possible that similar cases will be observed in the future. In such cases, the presence or absence of brain metastasis alone may no longer be a sufficient indicator of disruptions to the blood–brain barrier.

Naldemedine remains a useful drug for the treatment of constipation in patients taking opioids. However, in patients prescribed naldemedine, regardless of the presence or absence of brain metastases, the possibility of opioid withdrawal due to disruptions to the blood–brain barrier must be considered.

Better awareness of the side effects reported herein in association with the use of naldemedine, will contribute to further improvement in patient daily quality of life.
